# Genome-Wide Analysis of the Musa WRKY Gene Family: Evolution and Differential Expression during Development and Stress

**DOI:** 10.3389/fpls.2016.00299

**Published:** 2016-03-14

**Authors:** Ridhi Goel, Ashutosh Pandey, Prabodh K. Trivedi, Mehar H. Asif

**Affiliations:** ^1^Council of Scientific and Industrial Research-National Botanical Research InstituteLucknow, India; ^2^Academy of Scientific and Innovative ResearchNew Delhi, India

**Keywords:** abiotic stress, banana, differential gene expression, fruit ripening, WRKY gene family

## Abstract

The WRKY gene family plays an important role in the development and stress responses in plants. As information is not available on the WRKY gene family in Musa species, genome-wide analysis has been carried out in this study using available genomic information from two species, *Musa acuminata* and *Musa balbisiana*. Analysis identified 147 and 132 members of the WRKY gene family in *M. acuminata* and *M. balbisiana*, respectively. Evolutionary analysis suggests that the WRKY gene family expanded much before the speciation in both the species. Most of the orthologs retained in two species were from the γ duplication event which occurred prior to α and β genome-wide duplication (GWD) events. Analysis also suggests that subtle changes in nucleotide sequences during the course of evolution have led to the development of new motifs which might be involved in neo-functionalization of different WRKY members in two species. Expression and *cis*-regulatory motif analysis suggest possible involvement of Group II and Group III WRKY members during various stresses and growth/development including fruit ripening process respectively.

## Introduction

Transcription factors bind to the specific DNA motifs, regulate gene expression and control various signaling and regulatory networks involved in the proper growth and development as well as stress response in an organism. Out of numerous transcription factor gene families, WRKY gene family is known to be involved in various diverse processes in the plants from germination to senescence (Eulgem et al., 2000; Miao et al., 2007; Niu et al., 2012). Since the discovery of the first member of this gene family about 20 years ago, a number of members have been cloned and characterized from many plant species. In addition, with the advent of whole genome sequencing, the complete WRKY gene family has been analyzed in cotton (Dou et al., 2014), grape (Guo et al., 2014), populus (He et al., 2012), and many other plants.

This transcription factor gene family was named WRKY due to presence of highly conserved sequence WRKYGQK at N-terminus of the proteins (Rushton et al., 1995). In addition, members of this family also contain a conserved metal chelating zinc finger motif (C-X4–5-C-X22–23-H-X-H or C-X5–8-C-X25–28-H-X1–2-C) at the C-terminus of the WRKY motif. Members of this gene family regulate expression of genes through binding to the specific DNA sequence, W-box [(C/T)TGAC(T/C)], present in the promoter region of the target genes. In addition to the W box, WRKY domain also binds to a sugar responsive element, SURE, to regulate transcriptional expression (Sun et al., 2003).

In spite of the presence of highly conserved DNA binding domain, the binding of WRKY proteins to target genes varies due to the presence of variable number of WRKY domains and different pattern of zinc finger motifs (Rushton et al., 1995). Based on these differences, the WRKY genes are divided into three main groups (Eulgem et al., 2000). Group I and Group II WRKY members contain two and one WRKY domains respectively however; members from both the groups contain one zinc finger motif pattern of C2-H2 (C–X4–5–C–X22–23–H–X1–H). Group III WRKY genes contain one WRKY domain but vary in the presence of a different zinc finger motif pattern of C2-HC (C–X7–C–X23–H–X1–C). Based on the presence of different primary amino acid sequence, Group II WRKY genes are further divided into five subgroups (IIa, IIb, IIc, IId, and IIe; Eulgem et al., 2000).

Differential expression of the members of WRKY gene family and their involvement in various regulatory processes associated with biotic and abiotic stresses has been studied in various plants (Eulgem et al., 2000). In rice, *OsWRKY71* (Liu et al., 2007), *OsWRKY3* (Zhang et al., 2008), *OsWRKY 45-1*, and *OsWRKY45-2* (Tao et al., 2011) have been shown to be induced during bacterial pathogen attack. Similarly, WRKY genes like *AtWRKY8* (Chen et al., 2010)*, AtWRKY3* (Zheng et al., 2006)*, AtWRKY25* (Zheng et al., 2007)*, AtWRKY11*, and *AtWRKY17* (Journot-Catalino et al., 2006) are down-regulated under bacterial pathogen attack. Studies also suggest that *OsWRKY11* (Wu et al., 2009)*, HvWRKY38* (Xiong, 2010) *TaWRKY2*, and *TaWRKY19* (Niu et al., 2012) provide resistance to plants under drought stress. Members of the WRKY gene families are also known to regulate synthesis of the specific metabolites in response to abiotic stresses. Studies elucidated involvement of *OsWRKY11* in enhancing raffinose accumulation required for tolerance to plant against desiccation (Wu et al., 2009). In addition to the involvement to stress response and adaptation, involvement of WRKY genes has been demonstrated in the regulation of genes responsible for the proper growth and development of the plant. In *Arabidopsis*, studies suggest involvement of *AtWRKY6, AtWRKY22* and *AtWRKY53* in regulating senescence process (Robatzek and Somssich, 2002; Zhou et al., 2011; Miao et al., 2007). In rice, over-expression of *OsWRKY89* leads to reduction in the growth and internode length (Wang et al., 2007). *OsWRKY72* delays seed germination under normal conditions (Yu et al., 2010) and *AtWRKY7* was found to have a calmodulin (CaM)-binding domain (CaMBD) thus interacts with CaM (Park et al., 2005). Various studies suggest that alkaloid biosynthesis is regulated by members of WRKY gene family (Kato et al., 2007; Mishra et al., 2013; Agarwal et al., 2015). In addition to alkaloid, biosynthesis of the sesquiterpenes in cotton has also been demonstrated to be regulated by WRKY genes (Xu et al., 2004).

Therefore, it seems that WRKY genes play important role during plant growth, development and stress response. Studies suggest that during fruit ripening, expression of a number of genes related to stress response is enhanced (Kesari et al., 2007). However, involvement of WRKY gene family in fruit ripening has not been studied. The identification and functional characterization of the WRKY gene family from fruit crops will provide an insight into the regulatory aspects of biochemical and physiological processes operating during fruit ripening as well as response to various environmental stresses. Recently, the genome of two banana species; *Musa acuminata* (A genome) and *Musa balbisiana* (B genome) have been completely established (D'Hont et al., 2012; Davey et al., 2013). In this study, we have used these genomic resources to identify members of the WRKY gene family and correlated their expression with plant growth/development and stress response. Gene structure organizations and genome duplication events have been studied in detail using WRKY gene family from *M. acuminata* and *M. balbisiana* genomes. Their ortholog and homolog pairs have also been identified and their expression analysis was performed using available transcriptome datasets to identify involvement of specific WRKY gene family members in different processes.

## Materials and methods

### Identification and multiple sequence alignment

Protein coding (CDS) and whole genome sequences of two banana species, *M. acuminata* and *M. balbisiana*, were downloaded from banana genome database (http://banana-genome.cirad.fr). Hmmer-2.3.2 was used against the protein sequences of both the banana genomes to search the WRKY transcription factor encoding genes using plant Transcription Factor database (http://planttfdb.cbi.pku.edu.cn/; Jin et al., 2014) with *E*-value ≤ 10^−5^. All the identified WRKY genes were aligned separately against each other using multiple sequence alignment tool ClustalX2. The 60 amino acid conserved WRKY domain region was extracted from the sequences of identified proteins from *M. acuminata* and *M. balbisiana*. The WRKY genes which contain two WRKY domains, the domains were named as “C” or “N” depending on presence of domain at the C- or N-termini of the proteins respectively.

### Phylogenetic analysis

Due to the variable lengths of the complete protein sequences of the WRKY members, WRKY domains extracted from the identified proteins were used to draw the phylogenetic tree. *Arabidopsis thaliana* (85), *Oryza sativa* (121), *Vitis vinifera* (71), *Zea mays* (185) (Wei et al., 2012; Guo et al., 2014) and those identified from *M. acuminata* as well as *M. balbisiana* were used to construct the phylogenetic tree, making it a total of 778 non-redundant WRKY domains. The unrooted phylogenetic tree was generated using Maximum Likelihood algorithm with 1000 bootstrap value using JTT algorithm in MEGA 5.2 (Tamura et al., 2011). The phylogenetic tree generated was divided into the different groups on the basis of *Arabidopsis* and rice annotations and classification.

### Gene structure analysis

The gene structure information for each identified WRKY gene was extracted from the general feature format file of their respective genome and the gene structure images were generated using GSDS (Gene Structure Display Server; http://gsds.cbi.pku.edu.cn/; Hu et al., 2015) online server. The chromosomal images showing location of WRKY genes were generated using the MapChart program (Voorrips, 2002).

### Identification of motifs and tandem genome duplication

The conserve motif patterns were generated for the identified WRKY genes using MEME server (Bailey et al., 2009) with zoops models of minimum and maximum weight of 6 and 60 amino acid residues, respectively. These motifs were annotated using The Eukaryotic Linear Motif (ELM; http://elm.eu.org/) resource (Dinkel et al., 2014). Whole genome duplications were analyzed within the *M. acuminata* and *M. balbisiana* genomes using Musa ancestral blocks available at Plant Genome Duplication Database (PGDD; http://chibba.agtec.uga.edu/duplication/; Lee et al., 2013) and the visualization was carried out with CIRCOS (Krzywinski et al., 2009). The possible duplications between *M. acuminata* and *A. thaliana* as well as *M. acuminata* and *O. sativa* were also identified and visualized.

### Identification of *cis*-regulatory elements

Custom Perl script was used to extract the 1.5 kb upstream regions from the translation start codon of the genes and was considered as proximal promoter sequences. To identify *cis*-regulatory elements, PLACE *cis*-regulatory element online server (http://www.dna.affrc.go.jp/PLACE/; Higo et al., 1999) was used.

### *In silico* expression analysis

To identify the ripening related WRKY genes, expression levels in ethylene treated and untreated fruit transcriptome datasets of *M. acuminata*; dwarf Cavendish, genome AAA, var. Robusta, Harichhal (Asif et al., 2014b) was analyzed. Reads from both the datasets were mapped on the MaWRKY proteins using GSMapper (version 2.5.) and the TPM (transcript per million) and fold change for the annotated contigs were calculated. Ripening related expression of the identified WRKY genes was also analyzed using transcriptome datasets of *M. acuminata* fruit under acetylene response for 40, 60, and 90 days (D'Hont et al., 2012). In addition, to study expression of identified WRKY genes in response to biotic stress; root transcriptome datasets of *M. acuminata* under *Fusarium oxysporum* fsp cubense stress (Li et al., 2012) were used. The heat map for the annotated transcripts with their expression values were generated using MeV version 4.2 (Saeed et al., 2003).

To study the expression of the ortholog pairs of *M. acuminata* WRKY genes with the rice WRKY genes, the microarray cel files were downloaded for the rice development (GSE6893) abiotic stress (GSE6901) and heavy metal stress (GSE25206) condition. The microarray cel files were normalized using RMA in AffylmGUI package (Wettenhall et al., 2006) in R-bioC (ver 2.15) and normalized data was used for differential gene expression.

### Plant material and treatments

Dessert (*M. acuminata)* and cooking (*M. paradisiaca)* varieties of banana were used to study gene expression during development and fruit ripening. *M. paradisiaca* is the hybrid of *M. acuminata* and *M. balbisiana* belonging to AAB genome. Fruits from three independent plants of each cultivar at different developmental stages were harvested to collect the samples. Banana fingers from the same whorl of the hand representing similar developmental stage were treated with 100 μL/L ethylene for 24 h at 22°C in dark and then allowed to ripen in air as described in Lohani et al. (2004). Other vegetative tissues were harvested from 6 month old banana plants growing in the field. For RNA isolation, samples were quickly frozen in liquid nitrogen before extraction or stored at −80°C for further use.

### RNA isolation and expression analysis

Total RNA was isolated from banana tissues according to previously described protocol (Asif et al., 2006). Each RNA sample was treated with DNase I Digest kit (Sigma-Aldrich, USA) to eliminate DNA contamination. The integrity and size distribution of total RNA was analyzed by agarose gel electrophoresis. A NanoQuant (Infinite® 200 PRO NanoQuant, Austria) was used for RNA quantification. DNA-free RNA (5 μg) was used for synthesis of first strand of cDNA by using Revert Aid First Strand cDNA synthesis Kit (Fermentas, USA) as per manufacturer's recommendations. The quantitative RT-PCR was carried out with an ABI 7700 Sequence Detector (Applied Biosystems, USA) using SYBR Green chemistry. The amount of cDNA was normalized by using amplification of housekeeping banana *actin* as an internal control. Differential expression of five *M. acuminata* WRKY genes (*MaWRKY121, MaWRKY38, MaWRKY61, MaWRKY83*, and *MaWRKY119)* during fruit ripening was studied. Details of the oligonucleotide primers used in the study are provided in Supplementary Table 1. These MaWRKY genes were subjected to RT-PCR to validate their response in various stages of *M. acuminata* (AAA genome; dessert variety) and *M. paradisiaca* (ABB genome; cooking variety) during their course of ripening. The data from real-time PCR amplification was analyzed in terms of comparative fold expression following 2^−△△ct^ method. All the experiments were repeated using three biological replicates and the data were analyzed statistically (± Standard Deviation).

## Results and discussion

### Identification of the WRKY family members

The WRKY domain sequences were downloaded from Plant Transcription Factor database (http://planttfdb.cbi.pku.edu.cn/; Jin et al., 2014) and used to construct the HMM profile using HMMER (version 2.3.2). This profile was used for the identification of WRKY gene family members from the protein sequence of *M. acuminata* and *M. balbisiana*. After removal of partial and redundant sequences, a total of 147 and 132 WRKY genes were identified from *M. acuminata* and *M. balbisiana*, respectively (Supplementary Table 2). These MaWRKY and MbWRKY genes were named on the basis of their location on their respective chromosomes (Supplementary Table 2). Interestingly, though the WRKY genes are present on all the chromosomes, higher abundance was observed on chromosome 7 and 4 in *M. acuminata* while only in chromosome 7 in *M. balbisiana*.

There have been three rounds of genome duplication in Musa sp after the divergence of Musa from Zingiberales (D'Hont et al., 2012). Though, these duplication events resulted in the expansion of many gene families in Musa, there have also been simultaneous loss of many genes resulting in the control of gene family members (D'Hont et al., 2012). Some of the footprints of the genome duplication events were also observed in the WRKY gene family members. Our analysis suggests presence of duplicons in WRKY genes family members throughout the genome. The two Musa genome duplication blocks span chromosome 1, 2, 4 and 5, 8, 11, respectively. A total of 118 genes were present as duplication pairs throughout the genome, some of the genes had more than one duplicons.

### Classification of the WRKY family members

Based on the presence of the WRKY and Zinc finger domains, WRKY gene family members were classified into three groups. Group I WRKY gene family consists of two WRKY domains while Group II contains only one WRKY domain. Both these groups have same zinc finger motif pattern of C2-H2. Group III WRKY genes also contain one WRKY domain but with a different zinc finger motif pattern of C2-HC (Rushton et al., 1995; Eulgem et al., 2000). The *M. acuminata* and *M. balbisiana* WRKY genes were classified into these three groups on the basis on their similarity to *A. thaliana* (72) (http://www.arabidopsis.org/) and *O. sativa* (101) (http://systemsbiology.usm.edu/BrachyWRKY/WRKY/Rice.html) WRKY domains.

WRKY domains from 147 to 132 WRKY members from *M. acuminata* and *M. balbisiana* respectively were aligned with each other using multiple sequence alignment tool ClustalX2 (Supplementary Figures 1, 2). The identified WRKY members which contain two WRKY domains were named as “CTD” or “NTD” on the basis of presence of domain at the C- or N-termini of the protein respectively. This led to identification of 167 and 150 separate MaWRKY and MbWRKY domains, respectively.

**Figure 1:**
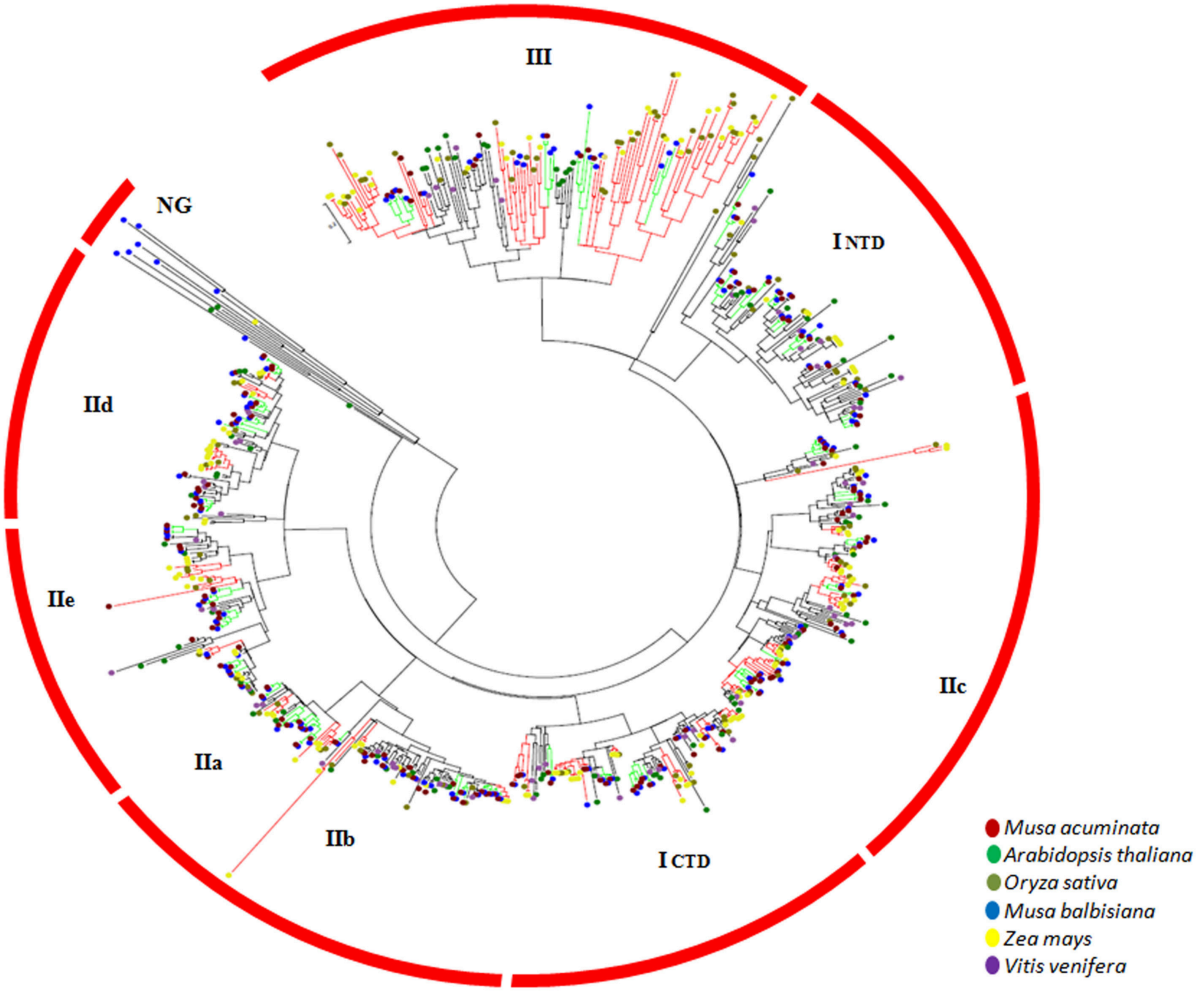
**Unrooted phylogenetic tree of WRKY domains**. The tree was constructed using WRKY domains from the complete WRKY gene families of *Musa acuminate, Musa balbisiana, Arabidopsis thaliana, Oryza sativa, Zea mays*, and *Vitis vinifera*. A total of 778 WRKY domains were used for the analysis. The unrooted phylogenetic tree was generated using Maximum Likelihood algorithm with 1000 bootstrap value using JTT algorithm in MEGA 5.2.

Further, the conserved 60 amino acid region of the WRKY domains was extracted from *A. thaliana, O. sativa, V. vinifera*, and *Z. mays* WRKY genes. This led to development of dataset comprising 778 non-redundant WRKY domains including *M. acuminata* and *M. balbisiana* WRKY gene domains (Table 1). An unrooted ML phylogenetic tree was constructed using these WRKY domains (Figure 1). Based on sequence similarity, the WRKY domains could be classified into Group I, II, and III genes. The Group II was further classified in Group II a-e. In *M. acuminata*, 24 genes were categorized in Group I whereas 106 and 16 identified as members of Group II and Group III, respectively. In *M. balbisiana*, 20, 87, and 21 WRKY genes were categorized in Group I, Group II, and Group III, respectively (Table 1). There were few WRKY genes (3 in *M. balbisiana* and 2 in grape) which were not assigned to any group and these were named as NG (not grouped).

**Table 1:** **Number of WRKY TF gene family members present in various monocot and dicot plants**.

**Groups**	**Subgroup**	***Arabidopsis thaliana***	***Musa acuminata***	***Musa balbisiana***	***Vitis vinifera***	***Oryza sativa***	***Zea mays***
Group I		13	24	20	12	18	36
	Group I-NTD	13	23	19	12	18	36
	Group I-CTD	13	21	20	12	18	36
Group II		46	106	88	39	50	82
	Group IIa	3	11	11	3	4	7
	Group IIb	8	21	17	8	7	11
	Group IIc	17	32	30	15	18	30
	Group IId	8	25	14	7	10	17
	Group IIe	10	17	16	6	11	17
Group III		13	16	21	6	35	31
NG				3	2		
Total		72	146	132	59	101	149

The Group II WRKY genes were further divided in five (a–e) subgroups. Analysis suggested that five MaWRKY genes (MaWRKY118, MaWRKY85, MaWRKY144, MaWRKY11, and MaWRKY99) were mis-annotated as Group IId in banana Genome hub on the basis of their annotation with *A. thaliana*. The phylogenetic analysis suggested that these WRKY genes are the members of the Group IIc. Therefore, the MaWRKY genes present in Group IIa, Group IIb, Group IIc, Group IId, and Group IIe contains 11, 21, 32, 25, and 17 members respectively. At the same time, 11, 16, 30, 14, and 16 genes were present in Group IIa, Group IIb, Group IIc, Group IId, and Group IIe, respectively in *M. balbisiana*. In Group I, all the C-terminal and the N-terminal WRKY domain were grouped independently (Figure 1).

WRKY genes formed monocot- and dicot-specific clades depicting that the WRKY genes have evolved simultaneously in both monocot and dicot plants from the common lineage (Figure 1). Interestingly, except for Group I NTD, phylogenetic analysis led to the formation of monocot-specific clades for all the groups. Group III had the largest monocot-specific clade comprising Musa-specific clades (Figure 1). There were few WRKY members which did not cluster in any of the three groups and were named Not Grouped (NG). Similar observation was also made in Setaria where such genes were included in Group IV (Muthamilarasan et al., 2015).

Within three groups of WRKY genes, there are four major lineages comprising Group IIa + Group IIb, Group IId + Group IIe, Group IIc + Group I (NTD and CTD), and Group III (Zhang and Wang, 2005; Rinerson et al., 2015). Till now, there were two proposed hypothesis regarding evolution of WRKY gene family in plants. According to Group I hypothesis, all the WRKY genes are evolved from the Group I-CTD (Zhang and Wang, 2005). In addition, alternative Group IIa+b hypothesis state that Group IIa and Group IIb evolved directly from the single domain ancestral algal WRKY genes separately from Group I WRKY genes (Rinerson et al., 2015). The phylogenetic analysis of the monocot and dicot plants favors the Group I hypothesis, stating that in the five plants selected, the evolution of the WRKY genes had occurred from the Group I ancestral WRKY genes (Figure 1).

### Duplication and Ks analysis

**Figure 2:**
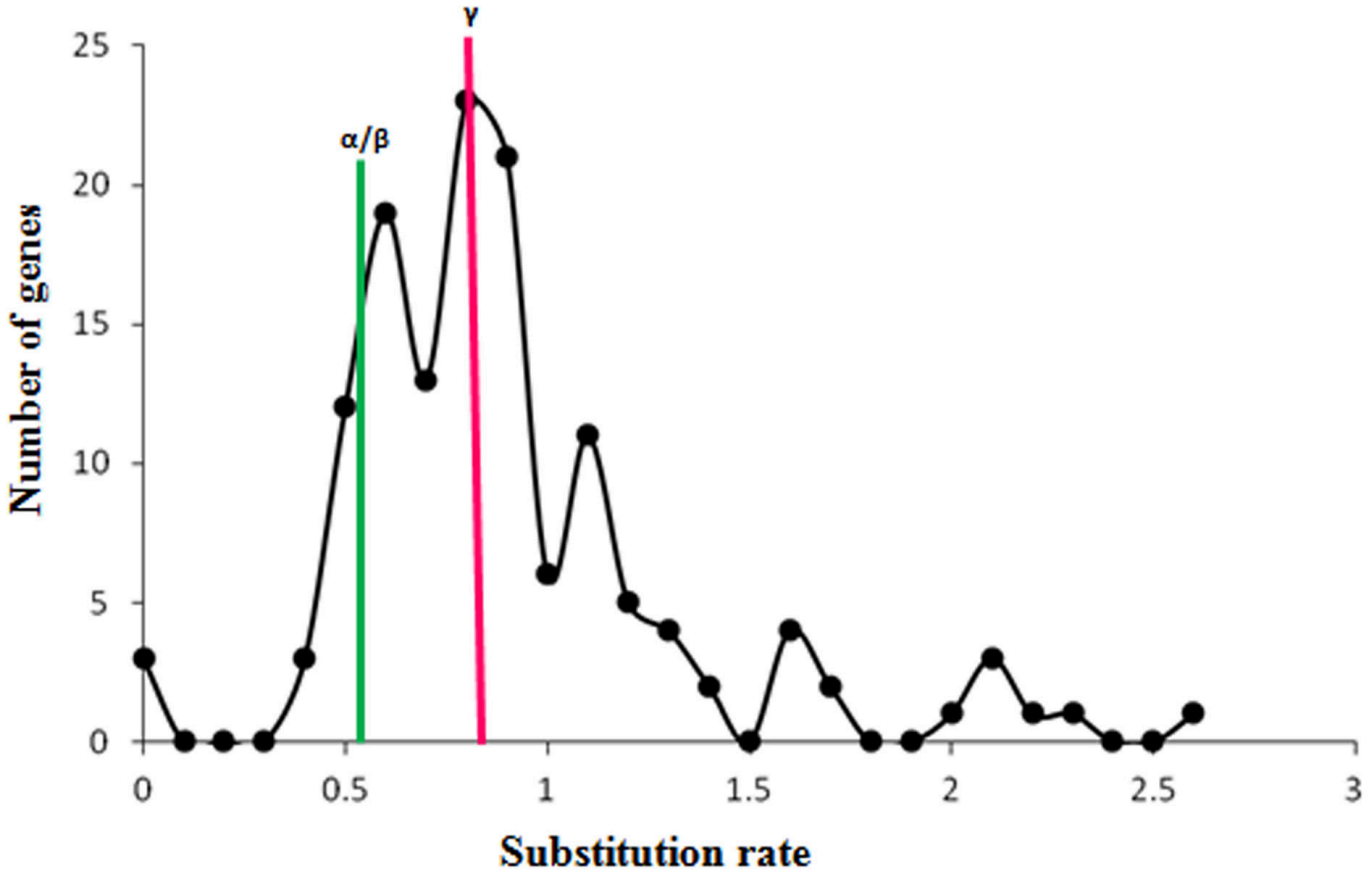
**Substitution rate of WRKY genes in ***Musa acuminate*****. The graph represents the ratio of synonymous (Ka) vs. non-synonymous (Ks) variations within the *Musa acuminate* WRKY gene family. X- and Y-axis represent the substitution rate and number of genes respectively. Green bars represent the substitution rate at the time α/β WGD and the red bar represents the substitution rate at the time of γ WGD.

The gene duplication pairs for *M. acuminata* were downloaded from the PGDD (http://chibba.agtec.uga.edu/duplication/) database and the genes containing WRKY domains were filtered. There were 136 WRKY gene duplication pairs which corresponded to 118 genes. This suggests that some of the WRKY genes have more than one duplicated genes (Supplementary Table 3). This could be due to multiple rounds of whole genome duplication events which have occurred in the Musa genome. The Ks (substitution rate) of these gene pairs was plotted against the number of gene pairs. Analysis revealed presence of two peaks corresponding to α/β and γ duplication events (Figure 2). It is interesting to note that more duplication pairs were present from the γ duplication event which could have occurred earlier. The analysis also suggests that recent duplicates of WRKY genes might have been lost due to the genome rearrangements (Figure 2). Interestingly, mapping of duplication pairs on the chromosomes suggested that many duplication pairs correspond to block 4 and block 7 of the Musa duplication blocks as suggested by D'Hont et al. (2012).

The orthologs of WRKY genes from *O. sativa* were also identified from the PGDD database. A total of 101 ortholog pairs corresponding to 62 MaWRKY genes were identified. The presence of additional 42 WRKY genes in *O. sativa* suggests that some genes have more than one orthologs (Supplementary Table 3). Analysis also suggests presence of 112 homolog pairs of WRKY genes between *M. acuminata* and *M. balbisiana*. Out of these, 111 and 112 were from *M. acuminata* and *M. balbisiana* WRKY genes, respectively. This suggests that there are specific WRKY genes which might have transferred and were conserved during the course of evolution within the Musaceae family (Supplementary Table 3). It was also identified that the ortholog genes within a chromosome of *M. acuminata* follow similar pattern as the *M. balbisiana* genome, depicting the conserved nature in the WRKY gene pattern.

### Gene structure and chromosome localization

The gene structures of the identified WRKY genes were retrieved from the genomic sequence of *M. acuminata* and *M. balbisiana*. The coding sequences of all the identified WRKY genes were mapped on to their respective complete genomes to identify intron-exon boundaries. The gene structures of the WRKY genes showed group-specific intron-exon patterns similar to *A. thaliana* and *O. sativa* (Wu et al., 2005). The number of exons varied from 1 to 9 in most of the WRKY genes of both the Musa species. Maximum number of exons, 23 numbers, was identified in *MaWRKY14* (Supplementary Table 4). In *M. balbisiana*, twelve genes contain more than nine introns and the numbers ranged from 11 to 25. It was also identified that a set of the WRKY genes in both the species contain an intron in between the WRKY domain as reported in *A. thaliana* and *O. sativa* (Wu et al., 2005).

It has already been reported that there are two types (R- and V-type) of introns which are present in between WRKY domain. R-type introns, the phase-2 introns, have a splice site directly on the R residue of the WRKY domain. On the other hand, V-type introns, phase-0 introns, have a splice site before the V residue, at the sixth amino acid after the second Cys residue, in the C2H2 zinc finger motif (Wu et al., 2005). In *M. acuminata*, 105 WRKY genes contain R-type intron while only 31 WRKY genes have V-type intron and 11 WRKY genes do not contain introns in between the WRKY domains. Similarly, in *M. balbisiana*, 88 and 25 WRKY genes contain R- and V-type introns respectively while 17 WRKY genes do not have any intron in between WRKY domain. Analysis suggested that V-type introns are present mostly in Group IIa and Group IIb while R-type introns are present mostly in all the other groups (Group I, IIc, IId, IIe, and III). WRKY genes which do not contain any intron in the WRKY domains, in both the genomes, are those WRKY genes in which domain are mostly present at the N-terminal of the group I. This loss of introns was considered as the result of intron turnover or due to reverse transcription of the mature mRNA followed by homologous recombination with intron-containing alleles (Wu et al., 2005; He et al., 2012). The positioning of the introns in between the WRKY domains was found in the ancestral WRKY genes. Thus, it can be hypothesized that the WRKY domains in the WRKY family member might have duplicated from the ancestral WRKY genes which contain introns, followed by the divergence rather than the formation of the similar genes through convergence events (Wu et al., 2005).

**Figure 3:**
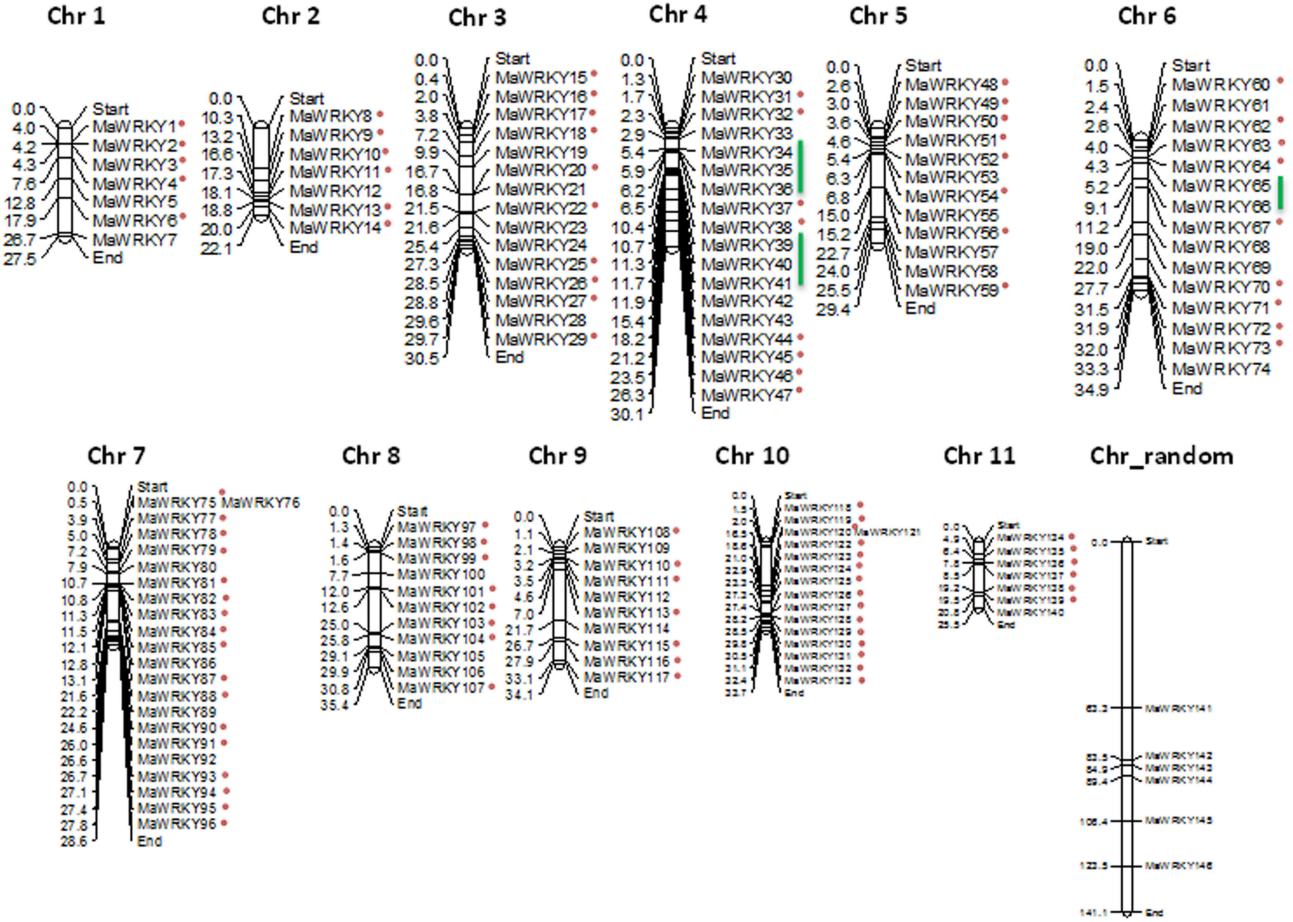
**Physical map of MaWRKY genes showing their chromosomal locations**. The genomic position of each identified WRKY gene was used to identify the chromosome localization on different chromosomes and the uncharacterized region. Vertical bars represent the chromosomes and numbers at the left indicate the position of genes (in Mb). The segmental duplicated genes are shown with red dots, and tandem duplicated with green lines.

The genomic position of each identified WRKY gene in both the genomes was also used to identify the chromosome localization. Analysis suggests that WRKY genes are distributed throughout the genomes of the *M. acuminata* and *M. balbisiana* (Figure 3 and Supplementary Figure 3). It was found that the maximum number of the identified MaWRKY (22) are localized on to the chromosome 7 followed by chromosome 4 and 10 with 18 and 16 MaWRKY genes, respectively. Six MaWRKY genes are present in the uncharacterized region of the sequenced *M. acuminata* genome (Figure 3). In *M. balbisiana*, maximum number (18) of the WRKY genes is present on the chromosome 7 while minimum (6) on chromosome number 1 and 2 (Supplementary Figure 3). Ten MbWRKY genes were present in the uncharacterized region of the sequenced *M. balbisiana* genome. It was observed that *MaWRKY75* and *MaWRKY76* on chromosome 7 as well as *MaWRKY120* and *MaWRKY121* on chromosome 10 are clustered together as the difference between them is 30 and 3 kb, respectively (Figure 3). Analysis also suggests that the identified MaWRKY and MbWRKY genes are evenly distributed on the different chromosomes and the uncharacterized region of both the genomes in accordance with the size of each chromosome (Figure 3 and Supplementary Figure 3).

### Unique motif analysis

**Figure 4:**
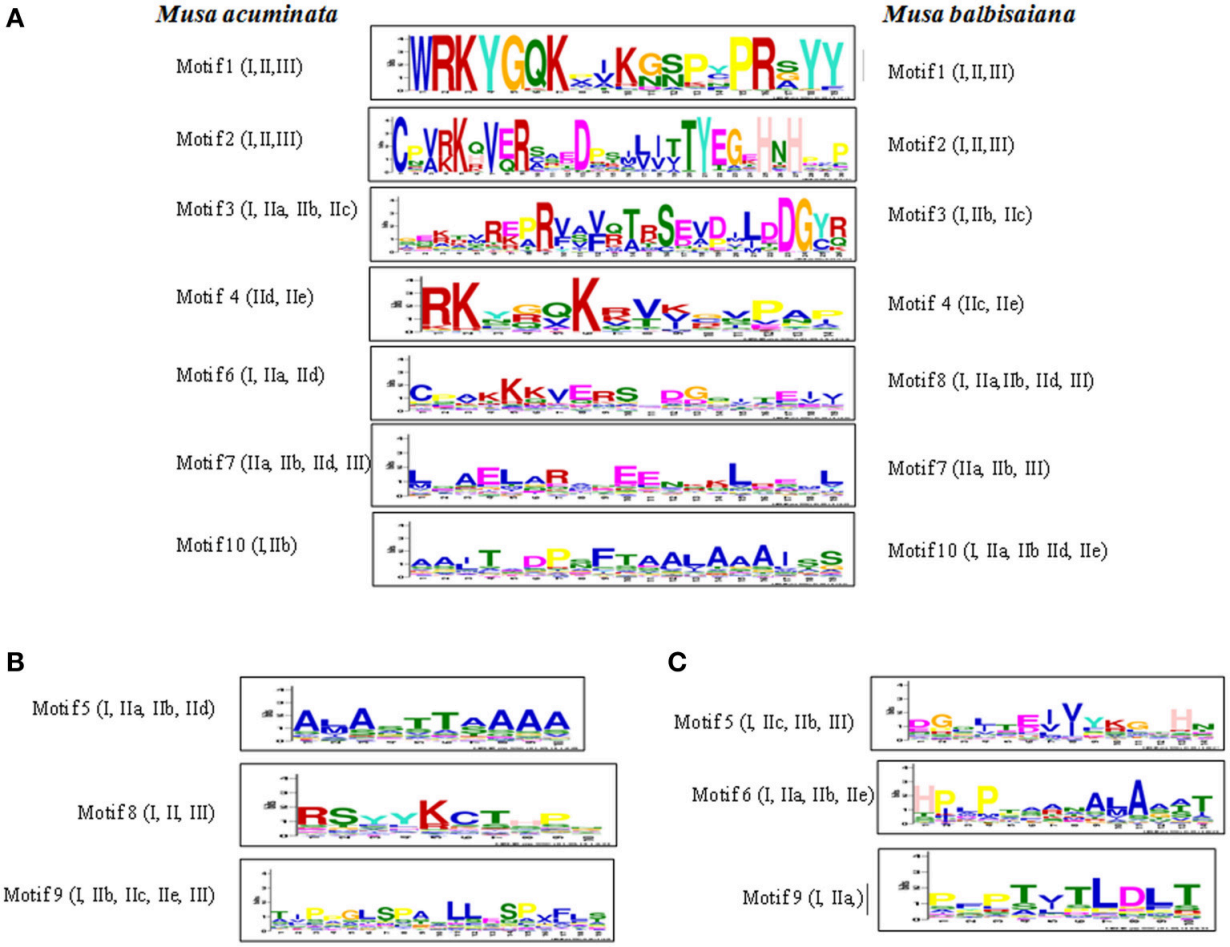
**Conserved motifs in WRKY proteins of Musa sp**. The conserved motifs were identified in 147 MaWRKY and 132 MbWRKY proteins using MEME server with zoops models of minimum and maximum weight of 6 and 60 amino acid residues, respectively. The motifs that were present in both the Musa sp are represented in **(A)**. Specific motifs that were present in only *Musa acuminate* are represented in **(B)** and those only in *Musa balbisiana* are represented in **(C)**.

Ten conserved motif patterns were identified for both *M. acuminata* and *M. balbisiana* (Figure 4) WRKY genes. Of these 10 motifs in WRKY members from both the species, motifs 1, 2, 3, 4, 7, and 10 were identical in *M. acuminata* and *M. balbisiana*. Motif 6 of *M. acuminata* was identical to motif 8 of *M. balbisiana* (Figure 4). Motifs 5, 8, and 9 of *M. acuminata* and motifs 5, 6, and 9 of *M. balbisiana* were species specific.

In *M. acuminata* and *M. balbisiana*, motif 1 and 2 are found to be conserved in all the identified WRKY genes while motifs 3 and 10 were conserved in Group I and few sub-groups of Group II WRKY members. Motif 4 was found to be kexin isozyme cleavage site while motif 10 is a pro-apoptotic protein. Motif sequence 1 is the basic WRKY domain of 20 amino acids which is found in all the WRKY genes. However, motif sequence pattern 2 is the variable sequence found in the zinc finger motif C2H2/C2CH (Figure 4). Motif 3 is the C2H2 conserve zinc finger motif which was found to be conserved at the N-terminal of the Group I WRKY genes (Figure 4). The Motif 6 of *M. acuminata* was similar to Motif 8 of *M. balbisiana* which is a fungi specific variant and was present in Groups IIa, IIb, IId, and III.

Some taxa-specific motifs were also identified between *M. acuminata* and *M. balbisiana*. In *M. acuminata* motif sequence 5 was found mostly in Group I and IIc while motif sequence 8 was present mostly in Group IIa and IIe. This motif is known to be responsible for the initiation of protein degradation. Motif 9 is phosphorylation recognition site present mostly in Group III, IId, and IIe (Figure 4). Likewise in *M. balbisiana*, motifs 5 and 6 are present on Group I, IIa, and IId while motif 5 is present in the Group IIb which is a phosphothreonine motif binding site. Motif 9 present in Group I, IIb, IIc, IIe, and III is a phosphorylation recognition site (Figure 4).

Apart from the basic 60 amino acid residue domain present in all the WRKY genes of different groups, there are differences in the WRKY domains present at the N and C terminal regions (Figure 4). These motifs are conserved in a particular group and thus might be involved in performing specific physiological and biological processes in plants. Therefore, it can be hypothesized that a specific group of WRKY genes might be responsible for the specific biological processes in plants (Kalde et al., 2003).

Analysis also revealed that the common motifs within *M. acuminata* and *M. balbisiana* WRKY members are the basic domain and the zinc finger motif. Also, the function of some of the motifs is similar in spite of the differences in their motif sequence pattern. Interestingly, Motif 9 present in both the Musa species have different sequence pattern but their function seems to be similar. This suggests that the function of the WRKY genes in a particular group is conserved and evolved simultaneously in specific taxa.

Apart from the basic WRKYGQK conserve motif in all the WRKY family members in *M. acuminata* and *M. Balbisiana*, WRKYGKK (in *MaWRKY23, MaWRKY80, MaWRKY91, MaWRKY106, MbWRKY42*, and *MbWRKY92*) and WRKYGRK (in *MaWRKY37* and *MbWRKY53*) motifs were also identified. These variations in the WRKY domains are also reported in *A. thaliana* (Dong et al., 2003). Apart from these motifs, *M. balbisiana* WRKY members also contain WRKYGNK (*MbWRKY59*), WRKYGHK (*MbWRKY54* and *MbWRKY104*), and WRKYGEK (*MbWRKY122, MbWRKY123*, and *MbWRKY124*) motifs (Supplementary Figure 1).

### *cis*-regulatory elements

**Figure 5:**
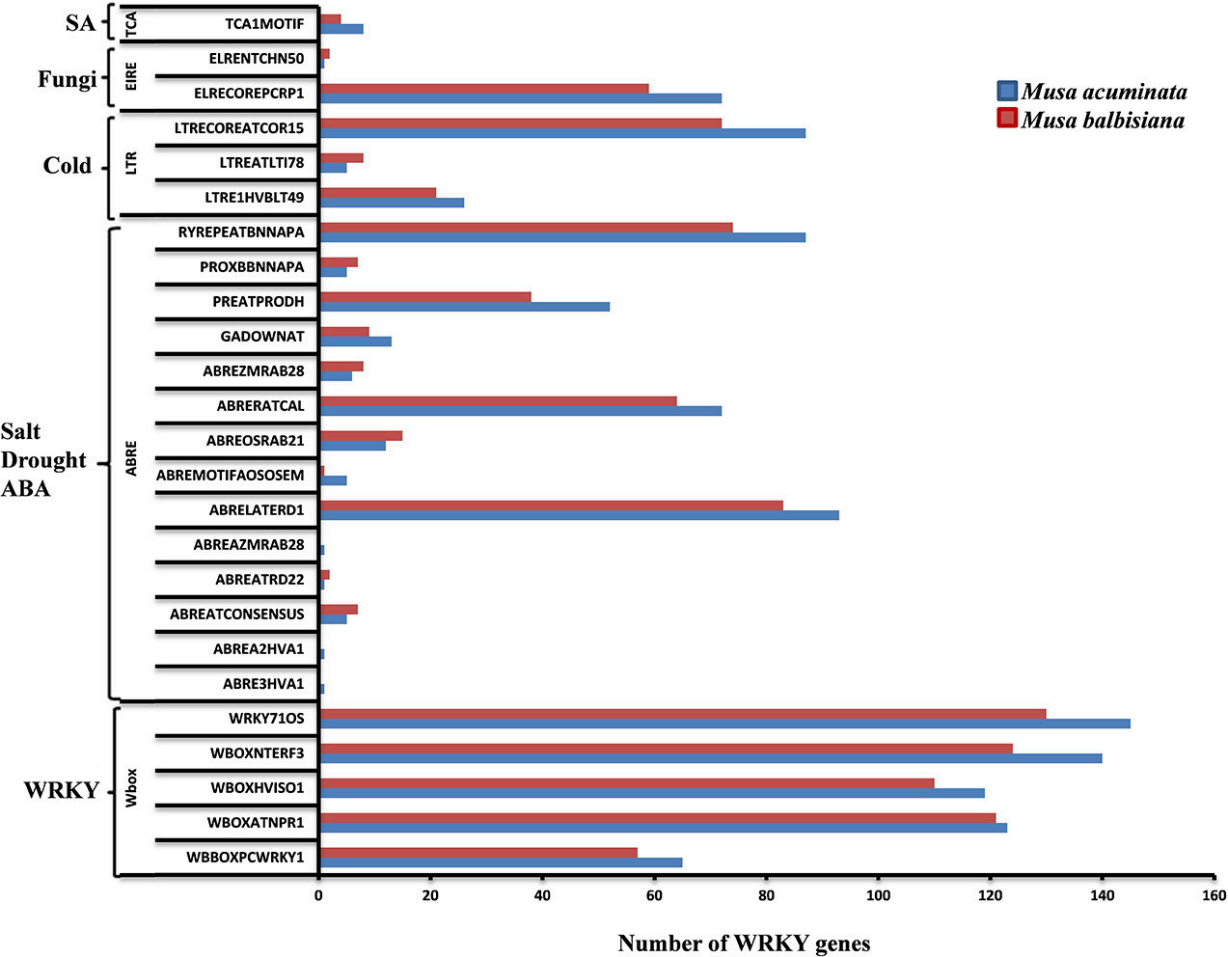
*****cis***-regulatory elements in WRKY genes of Musa sp**. The *cis*-regulatory elements were identified in the 147 MaWRKY genes and 132 MbWRKY genes, respectively, using the PLACE server. Custom Perl script was used to extract the 1.5 kbp upstream regions from the translation start codon of the genes and was considered as proximal promoter sequences. Graph was plotted on the basis of presence of *cis*-regulatory element responsive to specific elicitors/conditions (x-axis) in WRKY gene family members (y-axis). The blue bars represent *Musa acuminate* WRKY genes and the red bar represents the *Musa balbisiana* WRKY genes.

The 1.5 kb upstream regions of the WRKY genes were extracted and used for the identification of *cis*-regulatory elements using PLACE server and the *cis*-regulatory elements database. Many of the regulatory elements related to SA, cold, drought, ABA, and fungal response were identified in different genes. Interestingly, promoters from more than 50% of the WRKY genes harbor such elements indicating their important role during stress response (Figure 5). A set of WRKY genes also contain W-box and related motifs therefore regulation of these genes through other WRKY genes cannot be ruled out (Figure 5). A set of WRKY genes contain hormone-responsive elements of which majority of them are auxin and gibberellin response elements. A large number of genes also contain cytokinin, ethylene, and copper responsive elements (Figure 5). Previous reports have demonstrated that copper plays an important role in the signal transduction cascade of different hormones. A large number of genes also contain sulfur responsive element binding site which is known for essential for auxin dependent regulatory processes (Maruyama-Nakashita et al., 2005). Our analysis and previous studies (Knoth et al., 2007; Jiang and Deyholos, 2009; Levee et al., 2009) clearly suggest that WRKY genes play an important role during growth and development and stress responses. Interestingly, number of *cis*-regulatory elements identified in *M. balbisiana* is lesser as compared to those present in *M. acuminate* in the proximal promoter region used for the analysis (Figure 5).

The *cis*-regulatory elements identified, in this study, are evenly distributed throughout the promoter regions of the WRKY genes. The presence of SA, JA, ABA and ethylene related *cis*-regulatory elements suggest that these WRKY genes may be involved in various signaling pathways (Figure 5). The modulated expression of *AtWRKY18* (Chen and Chen, 2002)*, AtWRKY70* (Knoth et al., 2007), and *PtWRKY23* (Levee et al., 2009) has already been reported during fungi infection as well as in response to SA and JA. This suggests that a set of MaWRKY and MbWRKY genes might be involved in various defense related pathways. The other WRKY genes like *GsWRKY20* (Luo et al., 2013), *AtWRKY25*, and *AtWRKY33* (Jiang and Deyholos, 2009) *GmWRKY13, GmWRKY21*, and *GmWRKY54* (Zhou et al., 2008) were also found to be induced during abiotic stresses. In *M. acuminata*, Group I and Group III WRKY genes contains regulatory elements responsive to salt, drought, and ABA stresses while Group II WRKY genes contain regulatory elements responsive to cold stress and pathogen infection. In *M. balbisiana* the same pattern was also observed apart from Group II genes which contained elements responsive to salt, drought, and ABA stresses (Figure 5).

### *In silico* expression analysis

**Figure 6:**
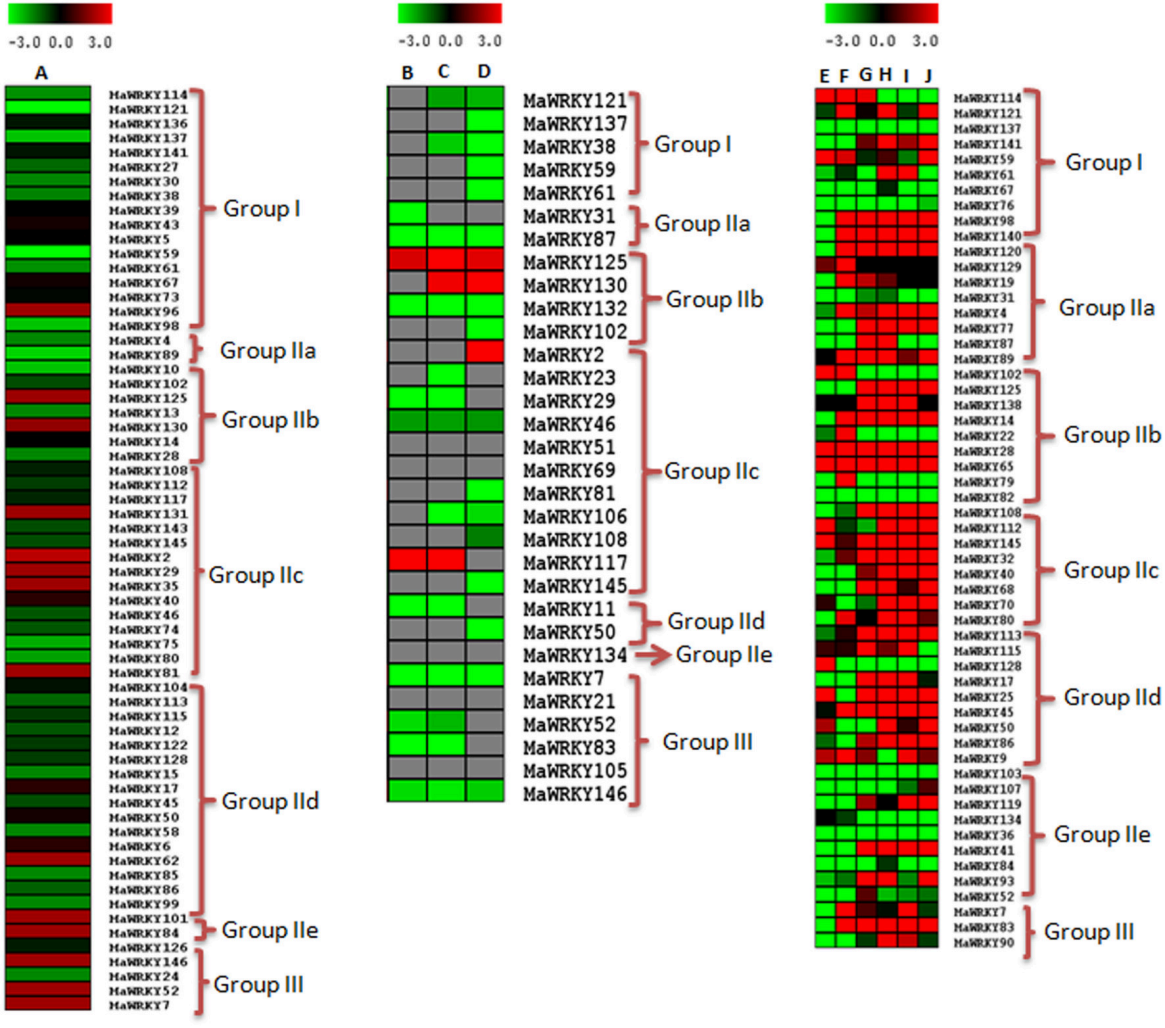
**Expression of MaWRKY genes in response the ethylene, acetylene, and fusarium infection**. To identify the ripening related WRKY genes, expression levels in ethylene treated and untreated fruit transcriptome datasets of banana (Asif et al., 2014a) was analyzed. Ripening related expression in response to acetylene exposure was analyzed using transcriptome datasets of *M. acuminata* fruit under acetylene response for 40, 60, and 90 days (D'Hont et al., 2012). Root transcriptome datasets under *F. oxysporum* stress (Li et al., 2012) were used to analyse biotic stress response of WRKY gene family. Log_2_ transformed count value are used for heat map construction. Panel **(A)** represents fold change between control (untreated) and ethylene treated (after 4 days of ethylene treatment. Panels **(B–D)** represent fold change between control (untreated) and acetylene treated after 40, 60, and 90 days, respectively. Panels **(E)**, **(G)**, **(I)** represent differential expression between control (untreated) and Foc1 treated after 3, 27, and 51 h, respectively. Panel **(F)**, **(H)**, and **(J)** represent differential expression between control (untreated) and Foc4 treated samples for 3, 27, and 51 h, respectively.)

WRKY genes are known to be involved in growth and development as well as in stress responses (Maruyama-Nakashita et al., 2005; Jiang and Deyholos, 2009; Levee et al., 2009). In this study, involvement of WRKY genes in fruit ripening has been studied using the transcriptome data set of *M. acuminata* (available at the banana genome hub; (D'Hont et al., 2012)) as well as our in house transcriptome dataset of ripe and unripe fruit (Asif et al., 2014b). Thirty-one WRKY genes showed differential expression in the transcriptome data of D'Hont et al. (2012) of which 20 genes showed increase in expression during ripening with a fold change of ≥ 1.5 (Figure 6 and Supplementary Table 5). The WRKY genes were also mapped to the transcriptome dataset of ripe and unripe fruit (Asif et al., 2014b). A total of 64 genes mapped successfully in ripe and unripe fruit transcriptome of *M. acuminata* of which 14 genes showed enhanced expression (fold change ≥ 1.5) during fruit ripening (Figure 6 and Supplementary Table 5). Analysis using both the datasets suggests that a set of WRKY genes are differentially regulated during ripening process in *M. acuminata*. Analysis revealed that during ethylene induced fruit ripening, Group I MaWRKY genes are down-regulated while a set of Group II MaWRKY genes and most of the Group III MaWRKY genes are up-regulated (Figures 6A,B).

To study the role of WRKY genes during biotic stress, WRKY gene expression was analyzed in transcriptome datasets related to *F. oxysporum* (Li et al., 2012) infection, available in public domain. It was observed that a large number (56) of WRKY genes expressed during *F. oxysporum* infected root tissue (Figure 6 and Supplementary Table 5). However, expression of WRKY genes was dependent on time duration post infection. There are 2 races (Foc1 and Foc4) of *F. oxysporum* which were studied at three time points (3, 27, and 51 h post infection; Figure 6 and Supplementary Table 5). While most of WRKY genes were up-regulated, decreased expression was observed for a set of WRKY genes post infection. Race specific differences in expression pattern were also observed as a set of WRKY genes expressed during early infection in Foc1 but not in Foc4 (Figure 6 and Supplementary Table 5). Expression of most of the WRKY genes continued to increase with time (Figure 6). During the pathogen attack in the *M. acuminata* plant, all the MaWRKY genes from Group II and Group III as well as some of the group I were up-regulated (Figure 6) suggesting response of specific banana genes during biotic stress response.

Since no public data is available to study the WRKY gene family expression during plant growth and development and other stresses in *M. acuminata*, the orthologs of the MaWRKY genes from *O. sativa* were retrieved and their expression was studied in available databases. Out of 147 WRKY genes in *M. acuminate*, only 62 had orthologs in rice database corresponding to 42 *O. sativa* WRKY genes (Supplementary Table 2). Analysis suggested that these WRKY genes are involved in the overall growth and development of the plant as well as in stress response (Supplementary Figure 4 and Supplementary Table 6). *O. sativa* WRKY genes were mostly up-regulated during abiotic stress (cold stress, drought stress, and salinity stress; Supplementary Figure 5A and Supplementary Table 6). In addition, data available in public domain (Chakrabarty et al., 2009; Dubey et al., 2010) was also used to study responsiveness of WRKY genes toward heavy metal stress. Analysis suggested that most of the WRKY genes are down-regulated during heavy metal stress (chromium, cadmium, arsenic, and lead; Supplementary Figure 5B and Supplementary Table 4). During the course of growth and development, Group I and III WRKY gene family members were up-regulated while Group II members were down-regulated (Supplementary Figure 4 and Supplementary Table 6).

In *M. acuminata*, most of the Group I WRKY genes were down-regulated during ripening and pathogen attack. Interestingly, their orthologos in *O. sativa* were up-regulated during abiotic stress and developmental processes (Figure 6, Supplementary Figures 4, 5). This suggests that the Group I WRKY genes were mainly involved in the developmental processes as well as in the abiotic stress response. Group IIc and Group IId genes are abundant in the set of WRKY genes regulating ethylene and acetylene related ripening. Our analysis suggests that in addition to involvement of Group II genes in fungal stress response, this group might also be responsible for ripening related gene regulation (Figure 6, Supplementary Figures 4, 5). Group III WRKY genes were found to be differentially regulated during early development of pollen and throughout the shoot development. Analysis also suggests Group III WRKY may not be involved in abiotic stress response (Figure 6, Supplementary Figures 4, 5).

The genes which showed differential expression under ethylene and acetylene exposure and fungal stress response had a high incidence of *cis*-regulatory elements related to elicitor response, ABREs and WRKY itself. This indicates that WRKY genes might be self-regulated by other WRKY members. Out of 147 WRKY genes, 26 genes contain the ethylene response element (ERELEE4). Out of these, 12 and 11 genes showed ethylene and acetylene responsive expression. Of these, five WRKY family members were commonly regulated by ethylene and acetylene.

### Validation of MaWRKY differential expression

The differential expression of five *M. acuminata* WRKY (*MaWRKY121, MaWRKY38, MaWRKY61, MaWRKY83*, and *MaWRKY119)* genes was analyzed during fruit ripening using qRT-PCR. Our *in silico* analysis using different datasets of *M. acuminata* (dessert variety) revealed that these WRKY genes are differentially regulated during fruit ripening. RT-PCR analysis suggested that expression of *MaWRKY121* is enhanced gradually during the banana fruit ripening process. In addition, *MaWRKY83* and *MaWRKY61* were late responsive as their expression enhanced significantly at the 6 and 8 day of ethylene induced fruit ripening respectively. No significant difference was observed in the expression of *MaWRKY38* during course of fruit ripening. This is in contrast to the down regulation of *MaWRKY38* gene in transcriptome analysis for ethylene and acetylene stress (Figures 6A,B).

**Figure 7:**
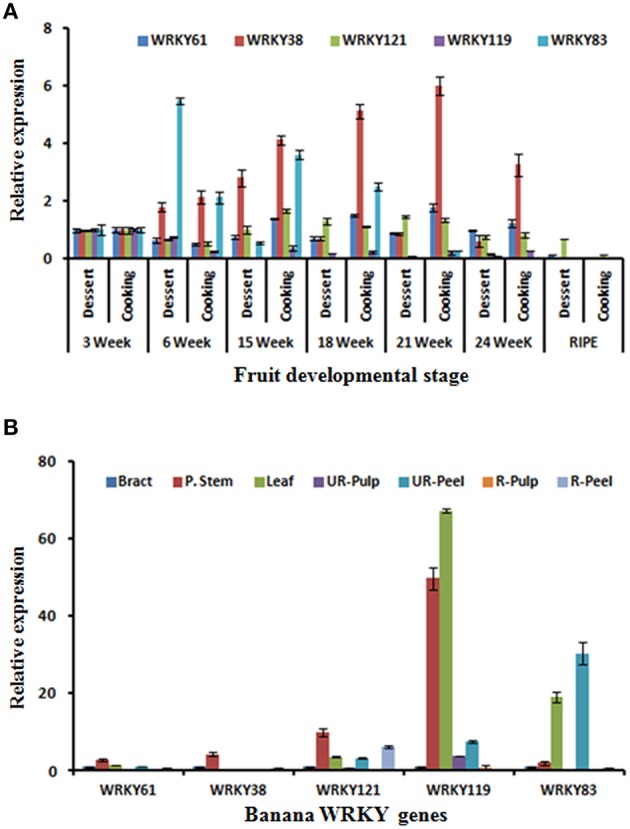
**Expression analysis of selected WRKY genes during fruit development stages, ripening, and in different tissues. (A)** Dessert (*M. acuminata*) and cooking (*M. paradisiaca*) varieties of banana were used to study gene expression during fruit development (in different weeks of development) and ripening **(B)** Expression analysis of WRKY genes in different tissues. Total RNA isolated from different tissues including Bract, Psudostem, Leaf as well as peel, and pulp tissues of ripe and unripe banana fruit was used for the analysis. The relative transcript abundance was normalized using banana actin gene.

Dessert (*M. acuminata)* and cooking (*M. paradisiaca)* varieties were also used to identify the possible role of banana WRKY genes in fruit development and ripening. These varieties have been used to study expression of members AP2/ERF and HDZIV gene families during fruit ripening (Lakhwani et al., 2015; Pandey et al., 2016). Expression analysis of five WRKY genes was carried out in different fruit developmental stages (3, 6, 15, 18, 21, and 24 weeks) and ripening stage of pulp (Figure 7). During fruit development, expression of *MaWRKY38* gradually increased in the cooking variety and was highest at 21 week. Distinctly, in dessert variety, expression was significantly lesser and gradually decreased after 15 weeks (Figure 7A). The significantly higher expression of *MaWRKY83* was observed at the 6 weeks in dessert variety (Figure 7A). This ripening related expression of *MaWRKY83* is similar to as observed during ethylene induced ripening (Figure 7A). The expression of *MaWRKY121* and *MaWRKY61* did not vary much during the course of ripening in both dessert and cooking variety.

The expression of selected MaWRKY genes was also studied at various tissues of the *M. acuminata*. It was observed that all the selected MaWRKY genes express mostly in stem as compared to other parts of the plant (Figure 7B). Highest expression of *MaWRKY119* gene was observed in stem and leaf while *MaWRKY83* gene was expressed mostly in leaf and unripe peel and was absent in unripe pulp (Figure 7B). Similar to WRKY gene family, genome-wide studies have been carried on few other gene families (Asif et al., 2014a; Lakhwani et al., 2015; Pandey et al., 2016) which has provided some inside about regulatory role of their members during fruit ripening and stress response.

In conclusion, this study suggests that WRKY gene family in *M. acuminata* and *M. balbisiana* is a large gene family. The gene family seems to have expanded before speciation, with minor changes in gene family after speciation. The expansion of the WRKY gene family was mainly due to the earlier γ duplication event of which most of the duplicons were lost in the recent α β duplication events. Our study suggests involvement of WRKY family members in various processes from development to ripening of fruit as well as biotic stress response in banana.

## Author contributions

MA and PT conceived and planned study. RG and AP carried out experiments. RG, AP, PT, and MA analyzed data. RG, MA, and PKT wrote the manuscript.

### Conflict of interest statement

The authors declare that the research was conducted in the absence of any commercial or financial relationships that could be construed as a potential conflict of interest.
