# Supplementary material for: Genome-Wide Analysis of the Musa WRKY Gene Family: Evolution and Differential Expression during Development and Stress
Source: Front Plant Sci. 2016 Mar 14;7:299. doi: 10.3389/fpls.2016.00299 (PMC4789551; doi:10.3389/fpls.2016.00299)
Supplement: Supplementary file 1 [file Presentation1.PDF]

Supplementary Information

Genome-wide analysis of the Musa WRKY gene family: evolution and differential expression during development and stress

Ridhi Goel[1,2], Ashutosh Pandey[1,#], Prabodh Kumar Trivedi[1,2,*], Mehar Hasan Asif[1,2,*]

[1]CSIR-National Botanical Research Institute (CSIR-NBRI), Rana Pratap Marg, Lucknow-226001, INDIA

[2]Academy of Scientific and Innovative Research (AcSIR), Anusandhan Bhawan, 2 Rafi Marg, New Delhi-110 001, India

[#]Present address (AP): National Agri-Food Biotechnology Institute (NABI), Department of Biotechnology, Government of India, C-127, Industrial Area, Phase VIII, S.A.S. Nagar, Mohali 160071, India

***Correspondence:**

MHA (mh.asif@nbri.res.in)

PKT (prabodht@nbri.res.in; prabodht@hotmail.com)

CSIR-National Botanical Research Institute (CSIR-NBRI),
Rana Pratap Marg,
Lucknow-226001, INDIA
Tel: 91-522- 2297958
Fax: 91-522-2205836, 2205839

Running title: Banana WRKY gene family

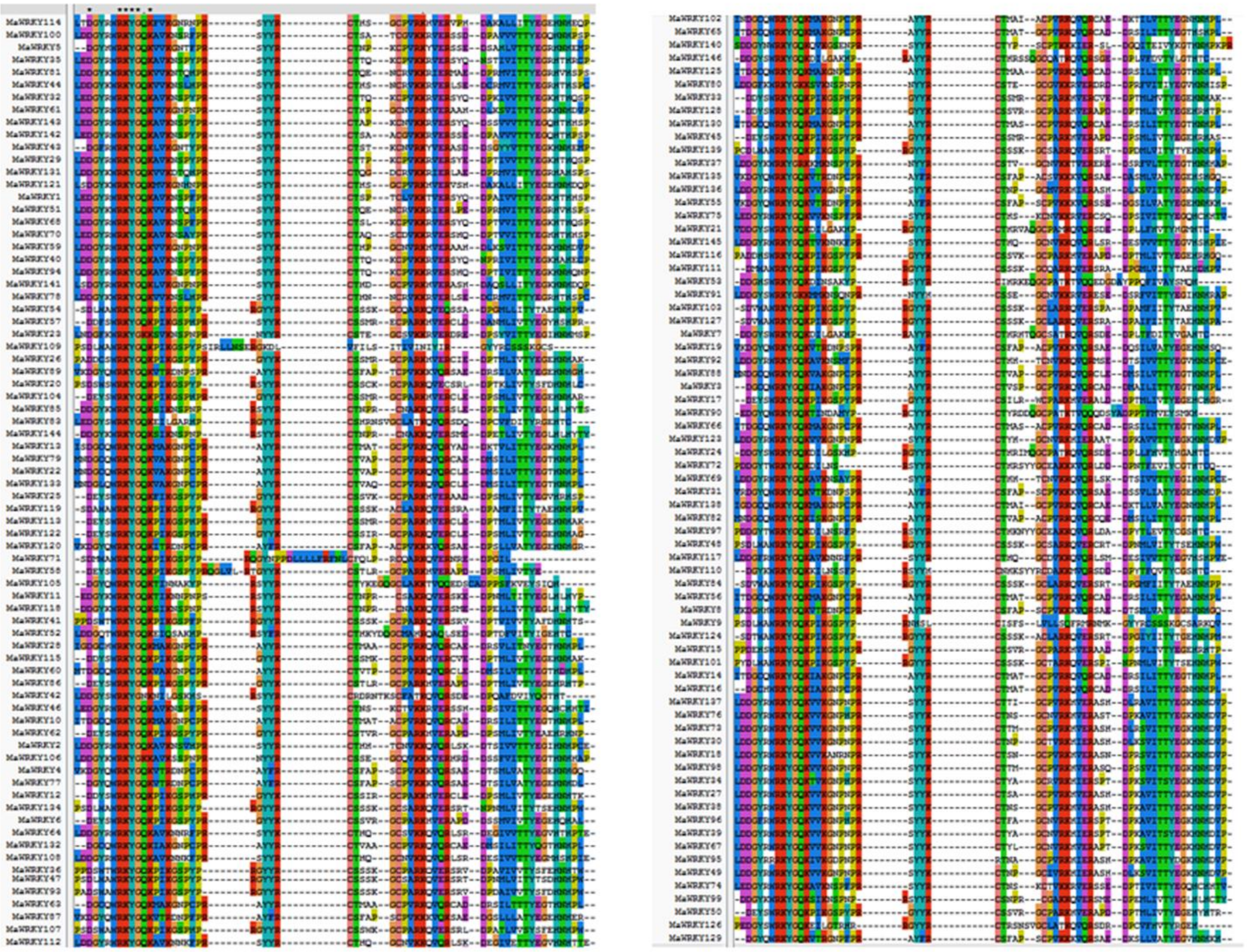

Supplementary Figure 1. Multiple sequence alignment of WRKY domains in *Musa acuminata* WRKY proteins. The coloured columns represent conserved amino acid residues.

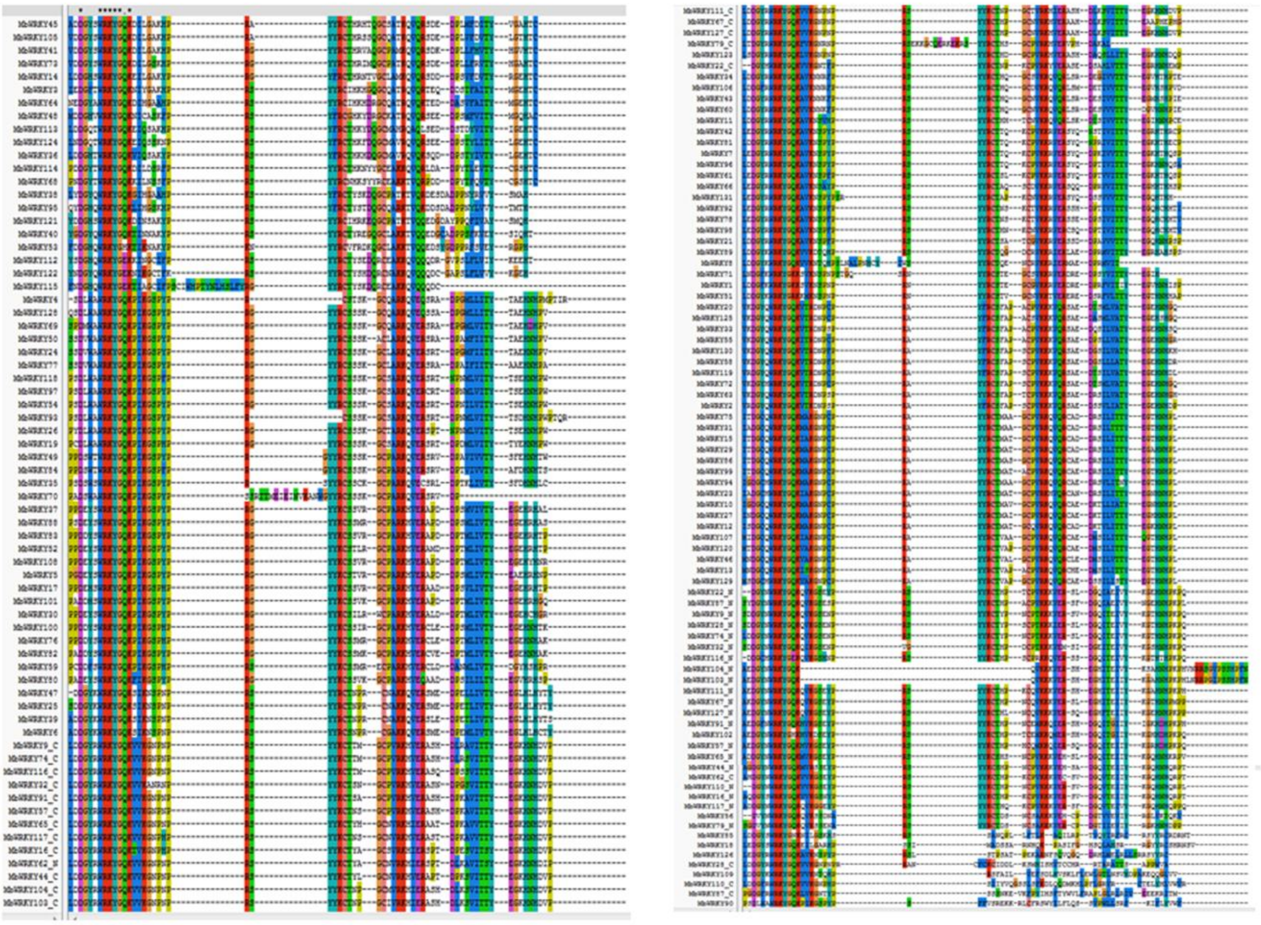

Supplementary Figure 2. Multiple sequence alignment of WRKY domains in *Musa balbisiana* WRKY proteins. The coloured columns represent conserved amino acid residues.

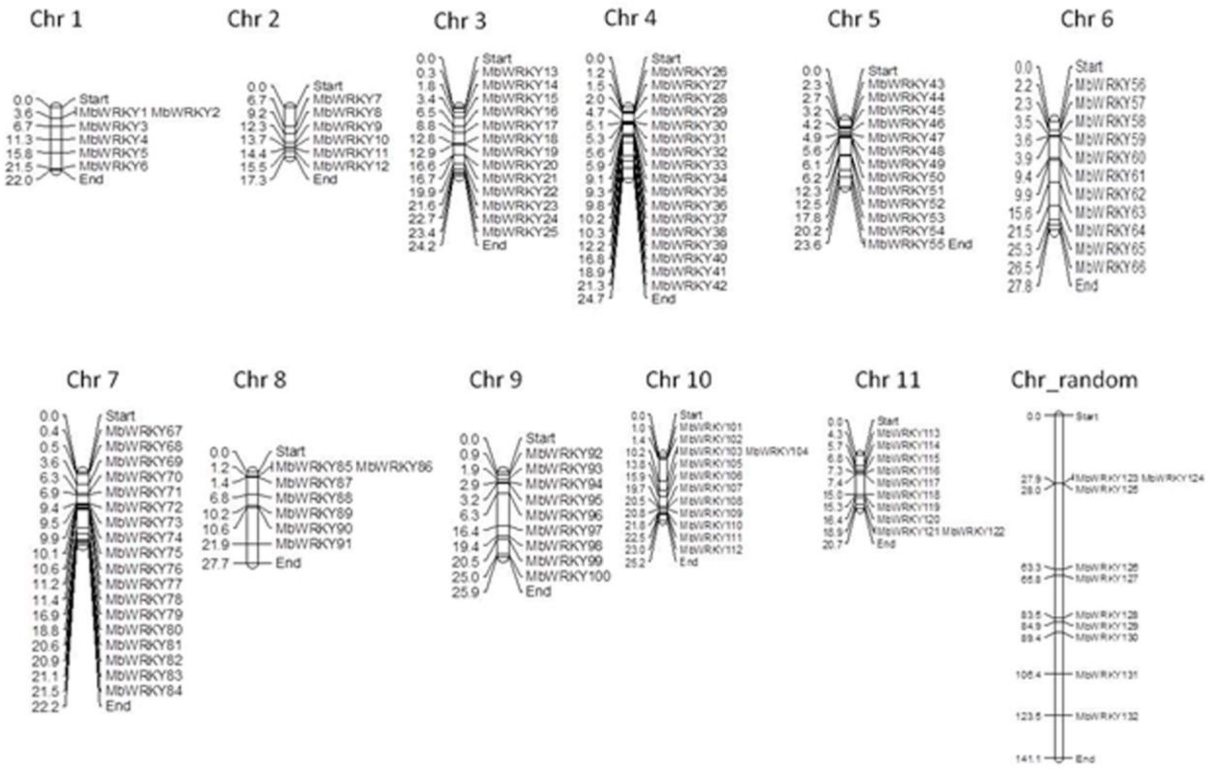

Supplementary Figure 3. Physical map of MbWRKY genes showing their chromosomal locations. Vertical bars represent the chromosomes and numbers at the left indicate the position of genes (in Mb).

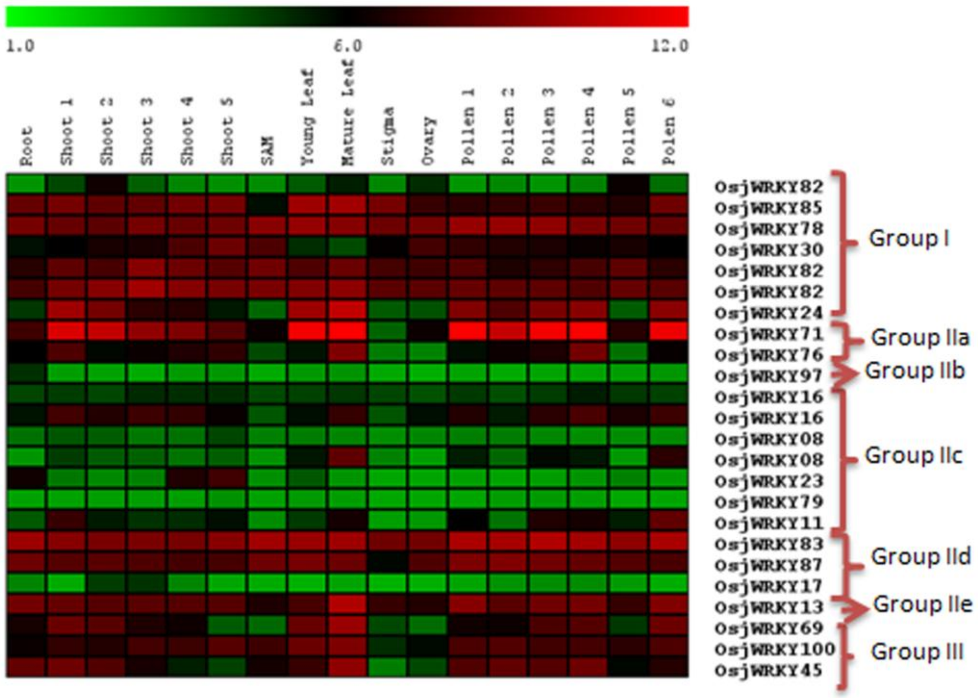

Supplementary Figure 4. Heat map of orthologs of MaWRKY genes from *Oryza sativa* during development. The orthologs of the MaWRKY genes from Oryza sativa were identified and their expression during various developmental stages. The scale bar at the top represents the fold change.

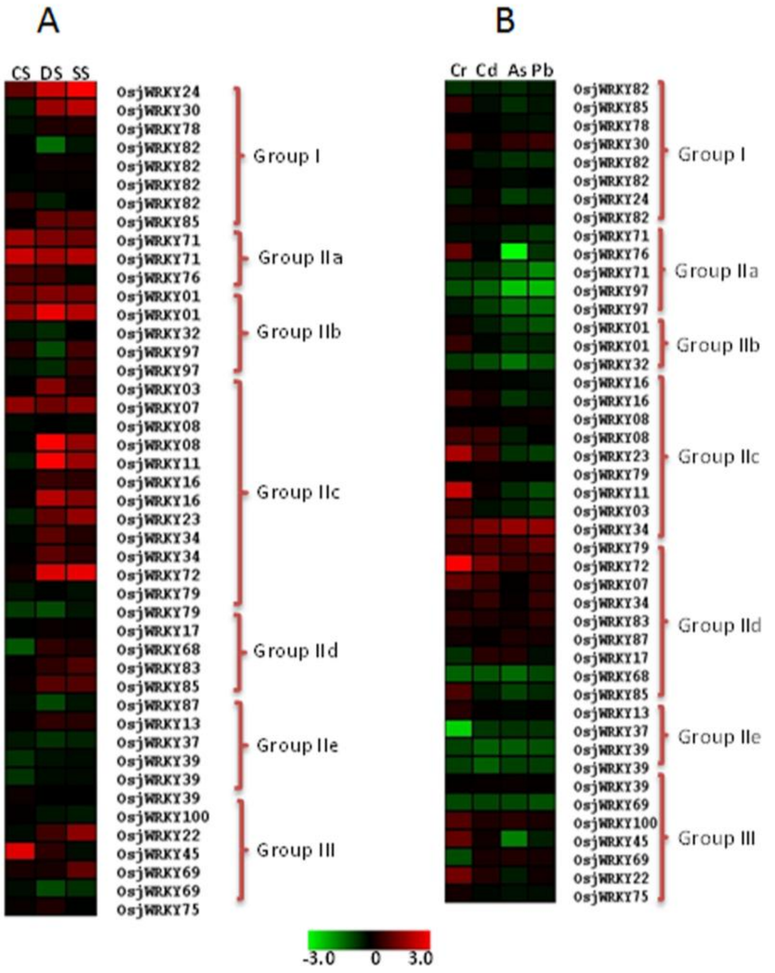

Supplementary Figure 5. Expression of orthologs of MaWRKY genes from rice during abiotic stress. The orthologs of the MaWRKY genes from *Oryza sativa* were identified and their expression during (a) cold, drought and salt stress and (b) during chromium, cadmium, arsenic and lead stress. For the analysis the microarray cel files available in public domain were downloaded for the rice development (GSE6893) abiotic stress (GSE6901) and heavy metal stress (GSE25206). The scale bar at the top represents the fold change.

Supplementary Table 1. List of primers used in qRT PCR

Gene	Forward Sequence (5' to 3')	Reverse Sequence (5' to 3')
MAWRKY38	GGAGTCAAAGCGCAGGAAGAT	GTGGCTCGCGGTTCATTTT
MAWRKY61	GAGGTTTGAAGGCCATGCA	GGTCTAAGCTGCTCCATTCCA
MAWRKY83	GGAGCCAGGCATCCAAGAG	GCAAGACATCCGACGCTGTT
MAWRKY119	GATGCTCACCGACCTCTTTCC	CACTTTCTGCTGCTGCTTCTTC
MAWRKY121	GTCTCGCATGATGCAAAAGC	GAGGTTGGCTGGTCATGATTG
MaActin	ATGACATGGAGAAGATCTGGCATCA	AGCCTGGATGGCAACATACATAGC
