# Supplementary material for: Genome-Wide Analysis of the Musa WRKY Gene Family: Evolution and Differential Expression during Development and Stress
Source: Front Plant Sci. 2016 Mar 14;7:299. doi: 10.3389/fpls.2016.00299 (PMC4789551; doi:10.3389/fpls.2016.00299)
Supplement: Supplementary file 2 [file DataSheet1.zip › Supplementary Table 1.pdf]

Supplementary Table 1. List of primers used in qRT PCR

Gene	Forward Sequence (5' to 3')	Reverse Sequence (5' to 3')
MAWRKY38	GGAGTCAAAGCGCAGGAAGAT	GTGGCTCGCGGTTCATTTT
MAWRKY61	GAGGTTTGAAGGCCATGCA	GGTCTAAGCTGCTCCATTCCA
MAWRKY83	GGAGCCAGGCATCCAAGAG	GCAAGACATCCGACGCTGTT
MAWRKY119	GATGCTCACCGACCTCTTTCC	CACTTTCTGCTGCTGCTTCTTC
MAWRKY121	GTCTCGCATGATGCAAAAGC	GAGGTTGGCTGGTCATGATTG
MaActin	ATGACATGGAGAAGATCTGGCATCA	AGCCTGGATGGCAACATACATAGC
